# Life-Cycle Assessment of an Industrial Recycling Process for Photovoltaic Panels Integrating Mechanical and Air-Assisted Pyrolysis Treatments

**DOI:** 10.1021/acs.energyfuels.6c00342

**Published:** 2026-04-16

**Authors:** Javier Ramirez-Cantero, Guillermo Garcia-Garcia, María Ángeles Martín-Lara, Zaida Hernández, Antonio Pérez, Mónica Calero

**Affiliations:** † Department of Chemical Engineering, Faculty of Sciences, 16741 University of Granada, Avda. Fuente Nueva, s/n, 18071 Granada, Spain; ‡ Greening Relive, C/Atalaya 4, 18015 Granada, Spain

## Abstract

The rapid growth of photovoltaic (PV) installations has increased the need for the sustainable management of end-of-life PV modules. This study proposes an integrated mechanical and thermal recycling system for crystalline-silicon PV waste, combining mechanical pretreatment for dismantling and material separation with air-assisted pyrolysis in a tilting furnace to remove encapsulant and backsheet polymers. This system recovers electronic components, glass, silicon, aluminum, copper, and a liquid oil suitable for energy recovery. An industrial-scale system capable of treating 60 PV modules per hour was assessed through life-cycle assessment to quantify environmental impacts, identify hotspots, and compare two scenarios: mechanical recycling alone versus combined mechanical and thermal recycling. Results show that the integrated recycling system has the greatest impact on photochemical ozone formation, climate change, and the use of fossil resources. The results for these impact categories make the thermal section responsible for over 61.7% of the total environmental impact, primarily due to the emission of ethyne, ethylene, carbon dioxide, and methane. Nevertheless, the production of valuable byproducts is a clear environmental advantage that largely compensates for the impact created by the integrated recycling system. A comparative LCA also concluded that integrated recycling reduces 21.3% of the single score of the mechanical recycling alone, confirming the environmental superiority of our proposed system. Overall, this study supports circular-economy strategies in the PV sector by demonstrating the environmental advantages of combining mechanical and thermal processes for industrial PV recycling.

## Graphical abstract

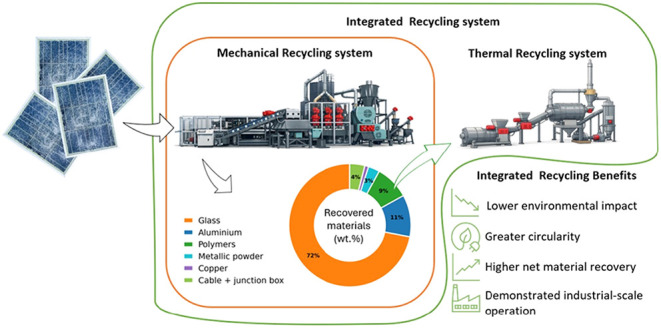

## 1 Introduction

The accelerating global transition toward low-carbon electricity systems has intensified interest in renewable energy technologies. Among these, solar photovoltaics (PVs) have emerged as one of the most mature and rapidly expanding options. Recent analyses forecast that despite a moderate deceleration PV installations will continue to increase between 2025 and 2030, with the Asia-Pacific region expected to account for nearly 80% of this expansion.^1–3^

Globally, the cumulative installed PV capacity surpassed 1.2 TW in 2023 and is projected to exceed 5 TW by 2035, driven by supportive decarbonization policies and continued cost reductions.^3^ However, the exponential deployment of PV technologies also entails a parallel challenge: the management of end-of-life (EoL) modules. Global PV waste generation is estimated to reach 60–80 million tonnes by 2050, positioning PV modules among the fastest-growing electronic waste streams.^4–7^

Crystalline-silicon modules dominate the global market, representing over 90% of the PV technologies. These composite devices consist primarily of glass (∼70 wt %), aluminum (∼10 wt %), polymers (∼10 wt %), and minor fractions of silicon, copper, silver, and tin. Their multilayer architecture, typically comprising a glass front cover, encapsulant (ethylene–vinyl acetate, EVA; or polyolefin elastomer, POE), silicon solar cells, and a polymeric backsheet, provides high durability and weather resistance but complicates material separation and recovery. Improper disposal practices such as shredding or landfilling can result in the loss of valuable resources and the release of hazardous substances, including fluorinated compounds from backsheets and metallic residues from solder alloys.^8,9^ Conversely, PV waste holds substantial recycling potential given its content of critical and valuable materials such as silver, silicon, aluminum, and glass.^10–12^ Recycling not only reduces the environmental impacts associated with landfilling but also supports resource recovery and circular-economy objectives.^13–17^

In Europe, policy frameworks have evolved to address PV waste management within the context of resource efficiency and circularity. The inclusion of PV modules in the Waste Electrical and Electronic Equipment (WEEE) Directive 2012/19/EU established Extended Producer Responsibility (EPR) obligations for collection and recycling. More recently, the Critical Raw Materials Act (CRMA) highlighted the strategic importance of elements such as silicon and copper, fostering recovery-oriented waste management across Member States.^18^ These legislative initiatives reflect a broader paradigm shift toward circular-economy principles, whereby efficient material recovery simultaneously mitigates environmental impacts and enhances resource security by reducing dependence on primary raw material extraction.

Current recycling technologies for PV modules can be broadly classified as mechanical, thermal, or chemical. Mechanical processes, including dismantling, crushing, and sieving, are cost-effective and widely implemented at the industrial scale but typically achieve low recovery rates for silicon, silver, and other metals due to incomplete removal of encapsulant and backsheet.^19–21^ Chemical processes, based on organic or inorganic solvents, enable selective recovery of metals and silicon but involve hazardous reagents and generate contaminated liquid effluents, limiting their scalability and environmental acceptability.^14,16,22^ Emerging alternatives, including high-voltage fragmentation and enzymatic delamination, have shown potential to enhance recovery rates and reduce environmental burdens, though their industrial maturity remains limited.^23–25^

Among thermal approaches, pyrolysis has been increasingly recognized as a promising technique for effective delamination and material recovery.^26^ This process involves the thermal degradation of encapsulant and backsheet (made of polymers) at moderate temperatures (400–550 °C) under controlled conditions, enabling the recovery of clean glass, silicon wafers, and metallic conductors while avoiding oxidation of valuable elements.^27–31^ Dias et al. (2016)^11^ demonstrated that pyrolysis at 500 °C for 30 min removes over 99% of polymers, facilitating recovery of silicon and glass. Similarly, Tao et al. (2023)^30^ developed a two-step pyrolysis process achieving near-complete recovery of backsheets, solar cells, and tempered glass. Nevertheless, the main challenges associated with pyrolysis remain: optimization of energy consumption, emission control, and process scalability.

Recent efforts have focused on integrated mechanical–thermal recycling systems, combining mechanical dismantling with thermal delamination. The mechanical stage facilitates the removal of external components (frames, junction boxes, and cables) and partial recovery of metals and glass, whereas the thermal stage ensures complete decomposition of the polymers and effective material separation. Air-assisted pyrolysis performed at 450–500 °C in tilting furnaces has demonstrated polymer-removal efficiencies exceeding 99% and glass-recovery yields above 95%, with favorable process safety and potential energy valorization of pyrolysis oils.^30,32^ The off-gases produced can be treated with calcium-based reagents to capture fluorinated species as stable CaF_2_ residues,^33^ while noncondensable gases may be combusted to supply process heat, thereby improving overall energy efficiency.

Despite promising technical outcomes, most studies remain confined to laboratory- or pilot-scale settings, and limited empirical data are available on the environmental performance of industrial-scale PV recycling. Existing Life-Cycle Assessments (LCAs) of PV recycling systems often rely on theoretical inventories or small-scale data,^12,34–40^ hindering consistent comparison across recycling routes and the formulation of evidence-based policies aligned with the EU’s circular-economy strategy.^41^

To address this, the present study conducts an LCA of an industrial-scale mechanical–thermal recycling line for crystalline-silicon PV modules. The system integrates mechanical dismantling with air-assisted pyrolysis in a tilting furnace operating at 450–500 °C. Our LCA quantifies the environmental impacts associated with the recycling process, identifies key environmental hotspots, compares two scenarios (mechanical recycling alone and an integrated mechanical-thermal recycling), and considers the environmental benefits of the byproducts generated.

## 2 Materials and Methods

This section first describes the industrial process performed to recycle EoL PV modules and then explains the first two phases of the LCA study: goal and scope definition and life-cycle inventory.

### 2.1 Description of the Recycling Process

The recycling process assessed in this study is based on an industrial line specifically designed for EoL PV modules, integrating mechanical and thermal operations within a continuous workflow. The industrial facility is capable of processing 60 PV modules per hour under controlled conditions. Figure 1 presents the overall process flow, which combines two complementary stages: a mechanical pretreatment and a thermal delamination treatment. Solid lines indicate the two main processing stages, namely, the mechanical treatment (upper section) and the thermal treatment (lower-left section). Material and energy flows between unit operations are represented by arrows. The dashed line defines the gate-to-gate system boundaries considered in the LCA. Recovered outputs include aluminum, glass fractions, metallic powders, copper, and pyrolysis products (oil subsequently separated into naphtha and kerosene). The equipment used to carry out the processes and the materials obtained from each step are explained below.

**Figure 1:**
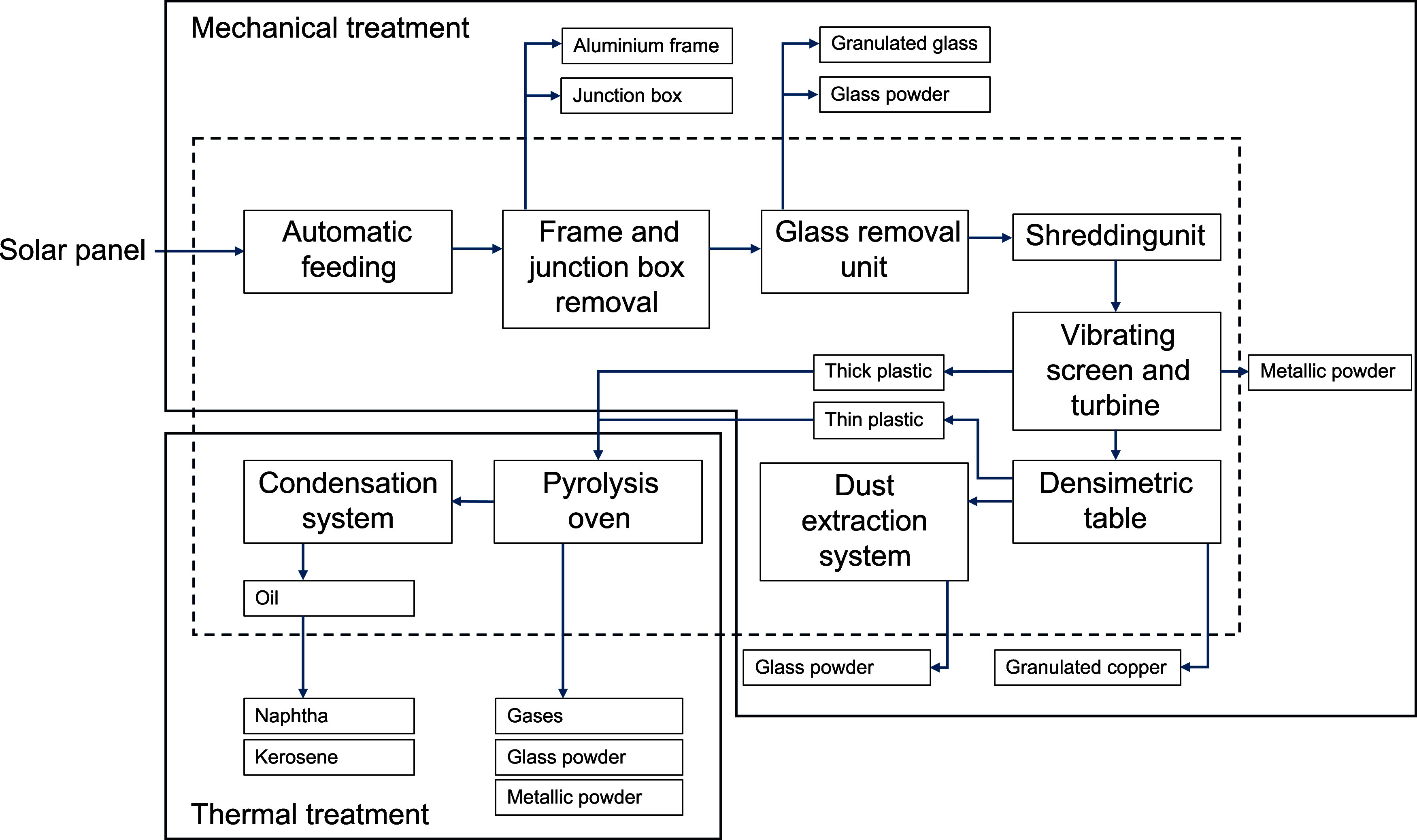
Block flow diagram.

The crystalline-silicon PV modules that enter the recycling system were manufactured by JA Solar. They are made of an aluminum frame, tempered glass, two layers of an EVA encapsulant, solar cells, a backsheet, and a junction box. The proportions of the materials used in a single 25-kg PV module are listed in Table 1. Although the values in Table 1 represent a standard crystalline-silicon module from JA Solar, it is acknowledged that silver (front grid) and silicon (cells) content can vary typically between 0.02–0.05 and 2.5–4.0%, respectively, depending on the manufacturer, cell technology, and year of production.^42–45^

**Table 1:** Composition of a PV Module

Component	Mass (%)	Mass (kg)
Aluminum	11	2.75
Total glass	72	18
Coarse particles	43.4	10.85
Fine particles	28.6	7.15
Total polymers	9	2.25
Encapsulant	5.4	1.35
Backsheet	3.6	0.90
Total metallic powder	3.03	0.757
Silicon	3	0.75
Silver	0.03	0.0075
Copper	0.97	0.242
Cable + junction box	4	1
**TOTAL**	**100**	**25**

The mechanical pretreatment involves a series of dismantling and size-reduction operations aimed at separating the external components of the modules and preparing the multilayer laminate for the subsequent thermal treatment. PV panels are automatically fed into the mechanical recycling system, which starts with the removal of the frame, junction box, and cables. The resulting laminate, composed of glass, EVA encapsulant, and backsheet polymers, silicon cells, metallic conductors, and the backsheet, is crushed and shredded to remove coarse glass particles and reduce material size. Next, a vibrating screen and turbine are used to separate the metallic powder (mostly made of silicon, with a very small proportion of silver and traces of aluminum and copper) and thick plastic. The remaining material is sent to a densimetric table to separate the granulated copper from the thin plastic. The remaining glass is removed via a dust extraction system. The materials recovered from the mechanical treatment are shown in Figure 2. The remaining composite fraction, which is mostly made of the polymers (thin and thick plastic) with traces of metallic powder and glass powder adhered to the plastic residues, is directed to the thermal section for complete delamination. Aluminum, copper from cabling, part of the glass, and part of the metallic powder are recovered as high-purity fractions suitable for direct recycling. In an alternative scenario, considered later in Section 3.1, in which these materials are not sent to thermal recycling, they are split between landfilling, incineration, and open burning, due to the difficulty of recycling them.

**Figure 2:**
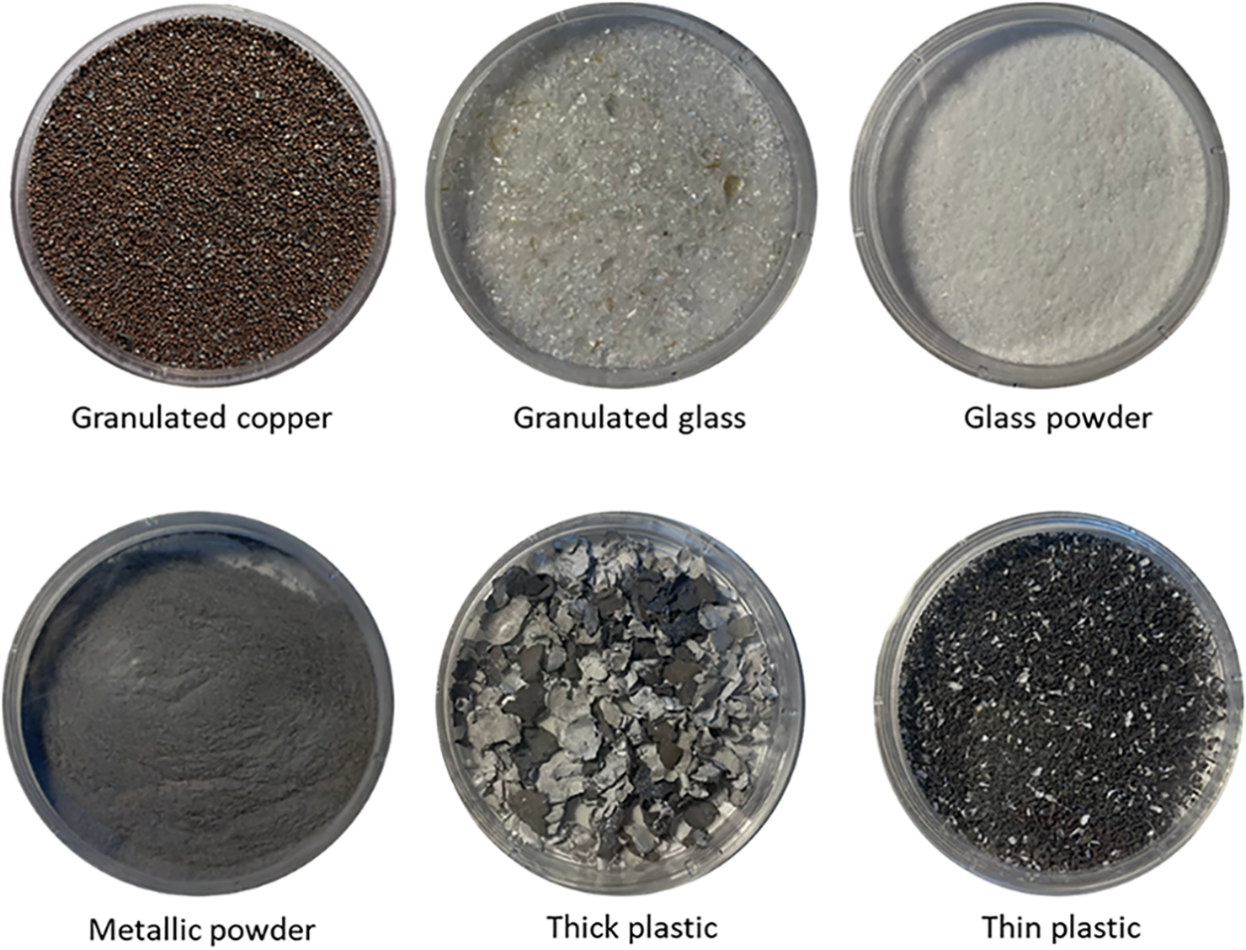
Materials recovered from the mechanical treatment.

The thermal stage consists of an air-assisted pyrolysis process carried out in a tilting furnace operating between 450 and 500 °C. The oscillating motion of the furnace ensures homogeneous heat distribution and gradual decomposition of the polymers, while a controlled flow of air promotes partial oxidation of organic residues without causing combustion of the inorganic layers. During this stage, the polymers are completely degraded and detached from the glass and metallic components, yielding three product streams:

I. A solid residue consisting of glass powder and a metallic powder mainly composed of silicon, with very small amounts of silver, aluminum, and copper.
II. A condensable vapor phase, collected as pyrolysis oil, composed mainly of naphtha and kerosene
III. A gas phase composed mainly of light hydrocarbons, CO, and CO_2_, with traces of fluorine and chlorine, which are currently emitted to the atmosphere

Overall, the integrated process achieves nearly complete delamination of PV modules, enabling the recovery of electronic components (cable and junction box), glass, silicon, aluminum, and copper, as well as a liquid oil fraction with potential for energy valorization or applications as fuel. The mechanical stage primarily contributes to the recovery of metallic and glass fractions, while the thermal treatment ensures the separation of the encapsulant and backsheet and fine materials. This combined configuration provides a comprehensive and scalable solution for PV waste management, maximizing material recovery while maintaining operational feasibility under industrial conditions.

### 2.2 Goal and Scope Definition

The goal of this study was to evaluate the environmental performance of the integrated mechanical-thermal recycling process for EoL crystalline-silicon PV modules and to identify the main hotspots within the process. This will be used to provide insights for process optimization and support a more circular management of PV waste.

This study followed an attributional LCA approach in accordance with ISO 14040 and ISO 14044 standards.^46,47^ In this way, the environmental impacts of the recycling process were quantified. Alternative scenarios where the recyclable outputs produced substitute similar materials were also considered and named as “AP” (scenario with “avoided products”), to also account for the environmental savings of removing the need to produce these products elsewhere. A gate-to-gate system boundary was adopted to make comparisons to other PV recycling plants easier. Processes within these system boundaries include all operations within the recycling facility, from the mechanical dismantling of modules to the recovery of secondary materials such as cables, glass, aluminum, silicon, metals, and oil, as shown in Figure 1. Upstream processes (module manufacturing and transport to the facility) and downstream stages (transport and reuse of recovered materials) were excluded, as the purpose of this study was to assess the recycling process. Nevertheless, the impact associated with material and energy inputs needed in the recycling process and that of treating the pyrolysis oil was accounted for.

The functional unit was defined as the treatment of one crystalline-silicon PV module weighing 25 kg, representing a typical commercial PV panel processed in an industrial line. All results were expressed per functional unit, allowing for comparison between scenarios and with previous LCA studies of PV waste management. This functional unit allowed for the recovery of 2.75 kg aluminum, 1 kg cable and junction box, 17.9 kg glass, 0.52 kg metallic powder (mostly silicon), 0.242 kg copper, 1.364 kg naphtha, and 0.260 kg kerosene. The environmental benefits of producing these products were accounted for in the AP scenarios.

The two sections of the recycling process were modeled as follows:

- Mechanical recycling (Section I), which includes aluminum frame removal, cable disassembly, and glass separation.
- Thermal recycling (Section II), which includes the subsequent delamination of the glass–polymer–cell laminate through air-assisted pyrolysis in a tilting furnace operating between 450 and 500 °C.

Additionally, an LCA comparison was made between the conventional mechanical recycling and the integrated mechanical-thermal recycling process recently implemented in the industrial plant, to make sure that the addition of the thermal process provides environmental benefits. To consider the same functional unit for both scenarios, the avoided products were considered in this comparison (AP scenarios). In the mechanical recycling scenario, the waste generated, comprised of the shredded encapsulant and backsheet with glass and metallic powder, mostly composed of plastic material, is split between landfilling, incineration, and open burning, as specified by the ecoinvent database for this difficult-to-recycle waste. In the integrated mechanical + thermal recycling scenario, this waste is the input material for the thermal recycling process. This has the advantage of eliminating the need to separate the encapsulant from the rest, which is currently a significant technical challenge.^48^

For each scenario, the results were calculated for each impact category, and the relative contributions of electricity, thermal energy, material inputs, and emissions were analyzed. Sensitivity tests were subsequently applied to evaluate the influence of process energy, for example, by switching to renewable energy, on the total impact profile.

This study relied on a combination of primary and secondary data. Primary data were obtained from two complementary sources. For the mechanical section, input and output flows were obtained from on-site measurements or derived from the technical datasheets of the equipment installed in the recycling line, including power ratings, throughput, and efficiency parameters. The inventory for the thermal section, consisting of polymer oxidation in a tilting furnace followed by the separation of naphtha and kerosene fractions, was developed by scaling up laboratory-scale pyrolysis results to industrial capacity. To ensure operational representativeness and avoid the pitfalls of simple linear magnification, this extrapolation integrated experimentally validated mass and energy balances with technical datasheets of industrial-scale equipment. Mass yields of the main product fractions were assumed to remain constant across scale, as they are primarily dependent on reaction chemistry and operating conditions (temperature, residence time), which were kept consistent with laboratory tests. However, energy requirements were not assumed to scale linearly. Additional energy penalties were incorporated to account for process inefficiencies due to heat losses and nonideal heat transfer, among other factors. Furthermore, to avoid overestimation of energy consumption under nominal conditions, a 75% loading factor was applied to the equipment’s rated capacity. This factor accounts for the fact that industrial machinery typically operates below its maximum power peak during continuous processes, reflecting more realistic electricity consumption.

The pyrolysis oil produced during the thermal treatment process consists of a mixture of naphtha and kerosene. We modeled the separation of these two products using fractional distillation. Taking into account their mass fractions, specific heats, and latent heat, 1385 kJ of heat is required for the separation, which is provided by steam. To condense the naphtha, 1023 kJ of heat needs to be removed. Considering a heat transfer loss of 10% and a water discharge temperature of 40 °C, this requires an input of 18.1 kg of water and consequently generates 18.1 kg of clean wastewater for discharge. To reduce water and energy use, it is recommended that this water be reused and that heat be recovered to supply the distillation boiler.

Finally, air emissions from the air-assisted pyrolysis experiments were collected in Tedlar sampling bags, which were connected to the reactor’s exhaust system. The sampling line was equipped with a condenser to remove moisture and condensable hydrocarbons from the gas before it reached the Tedlar bags. The collected gas samples were subsequently analyzed using an Agilent 990 Micro GC. An accredited external laboratory performed halogen analyses using the gas samples collected in Tedlar bags by gas chromatography coupled to mass spectrometry (GC–MS/GC–MSD-DRS), determining halogen-related compounds/indicators in the gas phase under standard calibration and quality procedures.

Secondary data were taken from the ecoinvent v3.11 (cut-off system model) database, ensuring the representativeness of background processes. For instance, electricity consumption was modeled according to the Spanish electricity grid mix for medium voltage to represent the supply conditions of the reference plant, thereby enabling comparison with previous studies based on similar assumptions. Long-term emissions were excluded but infrastructure was included in the assessment. This hybrid approach, which combines semiempirical industrial data with standardized background data sets, ensures methodological consistency, transparency, and adequate representativeness for assessing the environmental performance of the integrated recycling process.

A zero-burden approach was adopted, meaning that the incoming PV waste did not carry any environmental load from its manufacturing or use phase. This approach is common in waste treatment studies and isolates the environmental performance of the recycling process itself (a gate-to-gate approach, as described in Section 2.2). Allocation among byproducts was not applied, since the study focuses on the environmental load of the integrated recycling process, so byproducts can also be considered burden-free.

Environmental impact calculations were performed with SimaPro 10.2 software using the Environmental Footprint 3.1 (adapted) method, providing a comprehensive and transparent framework for interpreting the environmental profile of the recycling system. Characterization, normalization, weighting, and single-score results were calculated according to this method, which includes a wide range of environmental impact categories recommended by the European Commission.

### 2.3 Life-Cycle Inventory

The Life-Cycle Inventory (LCI) quantifies all material and energy flows associated with the recycling of one PV panel according to the functional unit defined in Section 2.2. The LCI combines primary industrial data from the mechanical recycling stage with pilot-scale data for the thermal treatment stage. The latter were extrapolated to an industrial scale using experimentally validated mass and energy balances to ensure representativeness. Primary data include the mass of PV modules processed, electricity consumption, and mass of recovered materials from both mechanical and thermal stages. Whenever possible, measurements were averaged over multiple operational cycles to reduce variability. Furthermore, all measurements were validated through cross-checks with material balances and operational logs. Secondary data were introduced only for upstream processes not directly measured in the facility, such as the Spanish electricity grid mix and heat provided by steam.

The main inputs of the system are the PV modules, electricity, thermal energy, and cooling water. The outputs comprise recovered materials (i.e., aluminum, cable, and junction box, glass, silicon, copper, kerosene, and naphtha), noncondensable gases, and wastewater. Table 2 presents the LCI, divided into two sections (i.e., mechanical recycling and thermal recycling).

**Table 2:** Life-Cycle Inventory

**Material or energy**	**Value**	**Unit**
**Mechanical recycling (Section I)**		
Inputs: material		
PV module	25	kg
Inputs: electricity		
Automatic feeding conveyor	1.75 × 10^–1^	kWh
Frame and junction box removal	1.25 × 10^–1^	kWh
Automatic feeding conveyor	1.75 × 10^–1^	kWh
Glass removal unit	3.00 × 10^–1^	kWh
Light conveyor for glass output	3.75 × 10^–2^	kWh
Medium feeding conveyor	3.75 × 10^–2^	kWh
Single-shaft shredder	3.94 × 10^–1^	kWh
Discharge screw conveyor	3.75 × 10^–2^	kWh
Impact turbine mill	6.94 × 10^–1^	kWh
Vibrating screen	1.38 × 10^–2^	kWh
Discharge screw conveyor (×2)	3.75 × 10^–2^	kWh
Densimetric table	4.38 × 10^–2^	kWh
Dust extraction system	3.25 × 10^–1^	kWh
Electricity grinder	6.88 × 10^–2^	kWh
Outputs: material		
Shredded encapsulant	1.35	kg
Shredded backsheet	0.90	kg
Glass powder	0.117	kg
Metallic powder	0.234	kg
Outputs: avoided products		
Aluminum	2.75	kg
Cable + junction box	1	kg
Granulated glass	10.85	kg
Glass powder	7.033	
Metallic powder	0.523	kg
Granulated copper	0.242	kg
**Thermal recycling (Section II)**		
Inputs: material		
Output material from Section I	2.601	kg
Cooling water	18.1	kg
Inputs: electricity		
Electric heating	6.87 × 10^–1^	kWh
Condensation system	1.75 × 10^–2^	kWh
Inputs: energy		
Heat for the distillation of pyrolysis oil	1385	kJ
Outputs: air emissions		
Halogen products	4.463 × 10^–5^	kg
Chlorine	4.463 × 10^–5^	kg
Hydrogen	9.013 × 10^–3^	kg
Methane	5.665 × 10^–3^	kg
Carbon monoxide	7.236 × 10^–2^	kg
Carbon dioxide	3.585 × 10^–1^	kg
Ethylene	7.434 × 10^–3^	kg
Ethane	1.132 × 10^–3^	kg
Ethyne	1.718 × 10^–1^	kg
Outputs: water emissions		
Wastewater	18.1	kg
Outputs: avoided products		
Light naphtha	1.202	kg
Medium naphtha	0.032	kg
Heavy naphtha	0.130	kg
Kerosene	0.260	kg
Metallic powder	0.234	kg
Glass powder	0.117	kg

## 3 Results and Discussion

This section presents the life-cycle impact assessment results and interprets these results to draw the conclusions of the study.

### 3.1 Impact Assessment

The Environmental Footprint 3.1 (adapted) method was used to transform the inventory data into quantifiable environmental impacts over a comprehensive range of impact categories.

The characterized results for the integrated recycling system, which includes the mechanical and thermal sections, are listed in Table 3. The contribution of the mechanical section dominates over the thermal section, accounting for 54.2–77.3% of the impact in each impact category, except for photochemical ozone formation and climate change, to which the thermal section contributes with 95.7 and 62.2% of the impact, respectively. The impact of the mechanical section is caused by the electricity used to power the equipment, 70% of which is attributed to the glass removal unit, single-shaft shredder, impact turbine mill, and dust extraction system. Therefore, optimizing the energy efficiency and/or use of this equipment is key to reducing the overall environmental impact of the integrated recycling system. Alternatively, using a greener source of electricity would also achieve lower impact results. This alternative is explored in Section 3.2.

**Table 3:** Characterization Results for the Integrated Recycling System

Damage category	Mechanical section	Thermal section	Total	Unit
Acidification	2.05 × 10^–3^	1.02 × 10^–3^	3.07 × 10^–3^	mol H^+^ eq
Climate change	5.01 × 10^–1^	8.24 × 10^–1^	1.33 × 10^0^	kg CO_2_ eq
Ecotoxicity, freshwater	6.31 × 10^–1^	3.01 × 10^–1^	9.32 × 10^–1^	CTUe
Particulate matter	9.98 × 10^–9^	7.25 × 10^–9^	1.72 × 10^–8^	disease inc.
Eutrophication, marine	4.20 × 10^–4^	1.90 × 10^–4^	6.10 × 10^–4^	kg N eq
Eutrophication, freshwater	7.89 × 10^–6^	4.22 × 10^–6^	1.21 × 10^–5^	kg P eq
Eutrophication, terrestrial	4.71 × 10^–3^	2.11 × 10^–3^	6.82 × 10^–3^	mol N eq
Human toxicity, cancer	1.09 × 10^–10^	5.52 × 10^–11^	1.64 × 10^–10^	CTUh
Human toxicity, noncancer	2.80 × 10^–9^	1.54 × 10^–9^	4.34 × 10^–9^	CTUh
Ionizing radiation	2.00 × 10^–1^	5.85 × 10^–2^	2.59 × 10^–1^	kBq U-235 eq
Land use	2.10 × 10 °	7.45 × 10^–1^	2.84 × 10^0^	Pt
Ozone depletion	8.57 × 10^–9^	7.24 × 10^–9^	1.58 × 10^–8^	kg CFC11 eq
Photochemical ozone formation	1.72 × 10^–3^	3.84 × 10^–2^	4.02 × 10^–2^	kg NMVOC eq
Resource use, fossils	1.62 × 10^1^	6.85 × 10^0^	2.30 × 10^1^	MJ
Resource use, minerals, and metals	1.72 × 10^–6^	5.41 × 10^–7^	2.26 × 10^–6^	kg Sb eq
Water use	5.04 × 10^–1^	1.48 × 10^–1^	6.52 × 10^–1^	m^3^ depriv.

In the thermal section, the main contributors for most impact categories are the electricity use, mostly to power the pyrolysis oven and heat for the distillation of pyrolysis oil. Therefore, optimizing the energy use of the pyrolysis oven is recommended for reducing the environmental impact of the thermal section. This could be achieved by insulating the oven more effectively to reduce heat losses or by reducing the temperature and/or residence time, provided this does not affect the pyrolysis outputs. Using alternative methods to provide heat that do not rely on electricity may also reduce the impact of this process. However, for photochemical ozone formation, the main contributors are the air emissions from the thermal process itself, particularly ethyne and ethylene, which account for 96.9% of the impact within the thermal section. Similarly, for climate change, the main contributors within the thermal section are the emissions of carbon dioxide and methane (63.8% for the thermal section). Overall, the climate change impact of the integrated recycling system is 1.33 kg CO_2_ eq.

The impact category most affected by the integrated recycling system is photochemical ozone formation, accounting for 74.3% of the total impact for normalized results and 54.4% for weighted results. The next most affected impact categories are climate change (8.6 and 27.8%, respectively) and use of fossil resources (8.3 and 10.6%, respectively). These differences are explained by the significant weighting given to climate change in the Environmental Footprint method. Overall, 38.3% of the single score for the integrated recycling system is allocated to the mechanical section, while 61.7% is allocated to the thermal section. This contrasts with the results for each impact category and is due to the larger normalized values obtained for photochemical ozone formation and climate change, which dominate the single scores.

Nevertheless, the environmental impacts caused by the integrated recycling system, listed in Table 3, allow not only for dealing with EoL PV modules but also for the production of valuable products like aluminum, reusable cable and junction box, glass, metallic powder, copper, naphtha, and kerosene. The functional unit defined in Section 2.2 includes the treatment of the EoL PV modules and the production of these materials. These products could replace similar products on the market, meaning that the impact of their conventional production could be subtracted from the impact of the integrated recycling system. A comparison of the attributional results presented in Table 3 with this alternative scenario can be seen in Figure 3. The impact results for the integrated recycling system are positive, meaning that they have an impact on the environment, whereas the results for the AP scenario are negative, meaning that the environmental impact is reduced due to the inclusion of the aforementioned avoided products. It can be seen that the reduction in environmental impact is significant for all impact categories. The avoided products that contribute most to this reduction in impact are silicon from metallic powder and cable and junction box, provided they can be reused. Overall, the integrated recycling system scenario achieves a single score of 0.134 mPt and the AP scenario a single score of −19.7 mPt, proving the environmental benefits of producing valuable byproducts through the proposed integrated recycling system.

**Figure 3:**
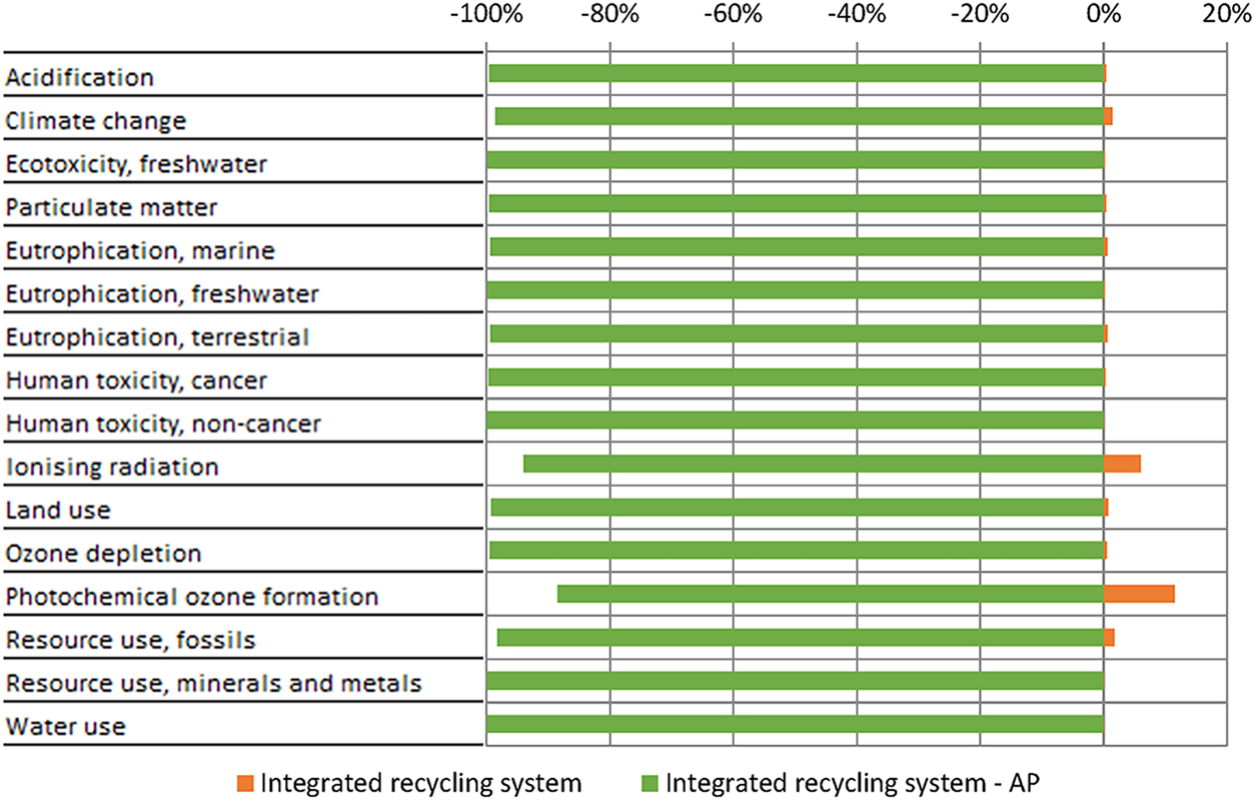
Comparison of characterization results for the integrated recycling system excluding and including the avoided products (AP).

To further confirm the environmental benefits of the proposed integrated recycling system, its impact results were compared with those obtained using the conventional system, which only employs mechanical recycling and disposes of the generated waste instead of pyrolyzing it. The same functional unit has been used for both scenarios, i.e., 25 kg of PV modules treated, including the production of avoided products, to make results comparable. The results are presented in Figure 4. The integrated recycling system achieves a lower impact for all of the impact categories. The maximum impact reduction is 32.9% for ozone depletion and 32.7% for the use of fossil resources. The impact remains virtually unchanged for the categories of use of mineral and metal resources. The impact reduction for the remaining impact categories is between these values. Overall, the single score for the integrated recycling system is 23.1% lower than that for the mechanical recycling system. In conclusion, integrated mechanical and thermal recycling of PV modules is recommended over mechanical recycling alone.

**Figure 4:**
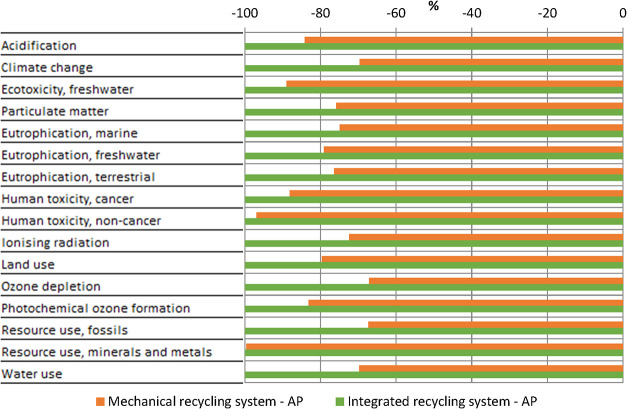
Comparison of the characterization results for mechanical and integrated recycling.

### 3.2 Interpretation

As demonstrated in the previous section, there are environmental advantages of implementing an integrated recycling system for PV modules that incorporates both mechanical and thermal recycling. Despite the significant environmental impact of the thermal section, the production of valuable byproducts that can be used as substitutes in the market provides a considerable environmental benefit. However, the environmental impact of the integrated recycling system remains high, partially due to significant electricity usage. Therefore, the geographical location of the recycling system and its electricity grid mix significantly influence the impact results. In this study, the Spanish grid mix was selected as the electricity source, as this is what powers the industrial plant modeled. For an alternative location with a high proportion of coal-fired power, such as Asia-Pacific countries, the environmental impacts would be significantly higher, particularly for categories such as climate change and fossil fuel usage. This must be taken into account when considering wider implementation of the technology described in this article, given that the Asia-Pacific region is expected to account for almost 80% of PV installation expansion, as mentioned in Section 1.

Figure 5 presents the results of a sensitivity analysis with an alternative electricity source. The impact assessment results for the integrated recycling system, with no avoided products, were calculated with the Spanish electricity grid mix and with electricity produced by a 570 kWp multi-Si PV open ground plant. These are both realistic sources of electricity for the industrial plant modelled. For this comparison, a low voltage was used in both scenarios to ensure a fair comparison with the same voltage electricity. Using PV electricity reduces the environmental impact in most categories. The reduction is most significant for ionizing radiation, with a reduction of 97.1%. The value for photochemical ozone formation is only reduced by 3.7%, but for climate change and use of fossil resources, the reductions achieve 31.0 and 78.3%, which is important since these three are the most affected impact categories according to the normalized results. The only increase in environmental impact is for ozone depletion and, mostly, land use due to the large area required to install the PV plant. Overall, switching to PV electricity can reduce the single score of the integrated recycling system by 33.8%. Another advantage is that when the PV modules powering the integrated recycling system reach their EoL, they can be replaced and treated directly in the same industrial plant.

**Figure 5:**
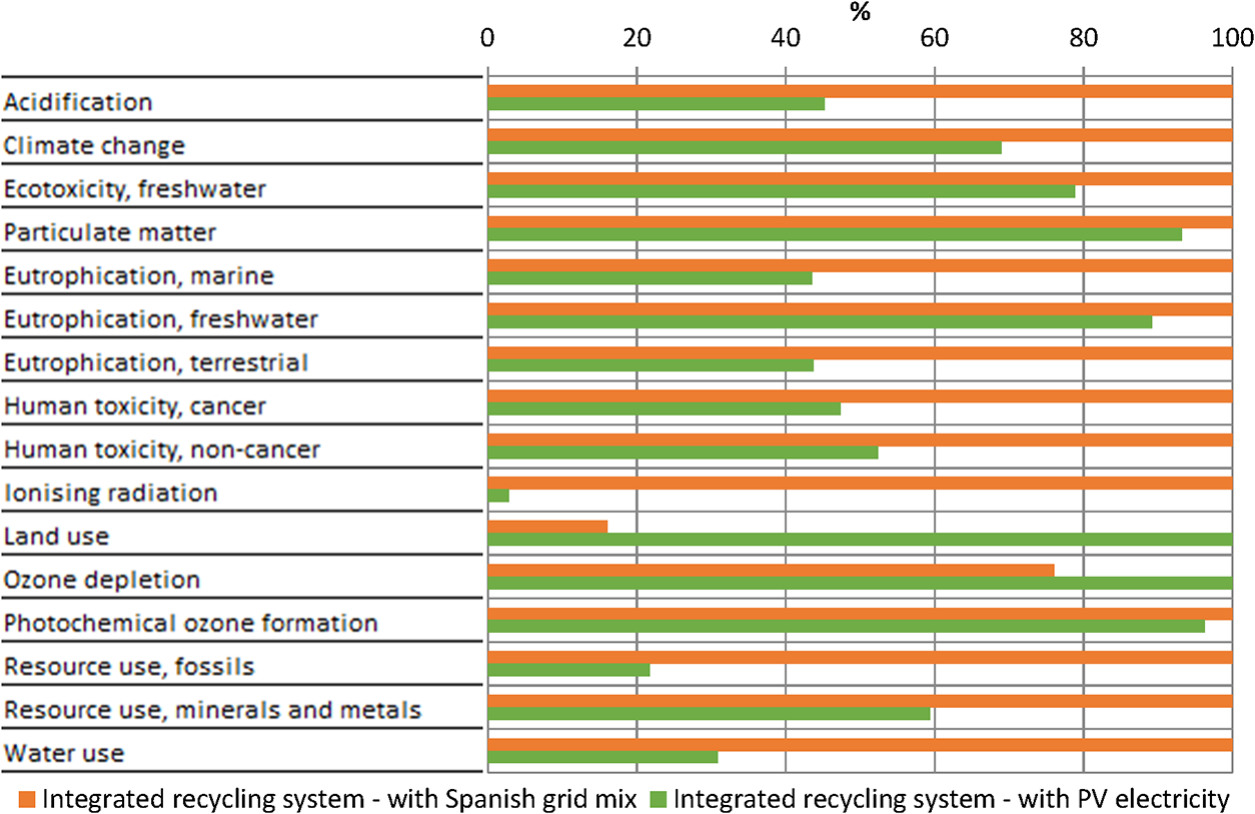
Comparison of the characterization results for the integrated recycling system powered by either the Spanish electricity grid mix or PV electricity.

In addition to the sensitivity analysis presented above, a basic uncertainty analysis was performed to test the robustness of the results. The Monte Carlo method^49^ was applied to the integrated recycling system. Avoided products were considered since their inclusion causes higher overall uncertainty. Lognormal distributions with a square geometric standard deviation of 10% for input parameters and 20% for output parameters were used. For the single score of this scenario, uncertainty results include a standard deviation of 9.89 × 10^–4^, a coefficient of variation of −4.75%, and a standard error of mean of 3.13 × 10^–5^. Out of the 1000 runs performed, 95% of the single-score results were between −22.8 and −18.9 mPt. These uncertainty results prove the robustness of the model and the reliability of the conclusions drawn.

In order to further reduce the environmental impact of the process, particularly that of the thermal treatment, we are currently investigating the filtration and neutralization of the pyrolysis gases to capture fluorinated species. We have achieved a reduction of halogens from 230 to 18.70 mg/Nm^3^ by reacting it with calcium hydroxide to produce CaF_2_, which has its own applications, for example, in analytical laboratories and in the production of hydrogen fluoride. We therefore recommend the installation of this filtration and neutralization system in the recycling line. Furthermore, using polymers with a low halogen content in the backsheet would be beneficial as it supports design-for-recycling strategies by simplifying end-of-life treatment, lowering emission control requirements, and enhancing the recyclability of recovered materials. Similarly, a fraction of the noncondensable gases could be combusted to supply some of the required heat, thereby improving the overall energy efficiency and reducing the need for heating.

The limitations of this study are primarily related to the choice of models, such as the selection of background processes and materials from the ecoinvent database, as well as the inherent subjectivity of the impact assessment methods. The reliability of the foreground data used to build the model is considered high, since most of it was directly measured in the industrial plant, was provided by the equipment specifications, or was scaled up from empirical laboratory experiments. It is however expected that the scaled-up pyrolysis process would have a lower environmental impact due to process optimization and the provision of heat by means other than electricity, for example, via an industrial boiler. The only step modeled and not performed yet in the recycling system is the separation of naphthas and kerosene, which has been modeled using mass and energy balances.

The first recommendation from this study is to use mechanical and thermal recycling of PV modules instead of mechanical recycling alone. In order to reduce the environmental impact of the integrated recycling system, the energy consumption of the pyrolysis oven must be closely examined. It is recommended to reduce heat losses through better insulation, to minimize the temperature and/or residence time (if this does not affect the pyrolysis outputs), and the use of a more efficient heating method than electric heating. For all processes powered by electricity, switching to PV electricity would significantly reduce the environmental impact of the integrated recycling system. We also recommend installing a filtration and neutralization system to capture fluorinated species or, alternatively, using polymers with a low halogen content in the backsheet if possible. Finally, the noncondensable gases should be combusted to reduce the need for heat and remove the emissions of pollutants like ethyne, ethylene, and methane, which significantly increase the impact of photochemical ozone formation and climate change.

Given the variability in PV panels, recycling technologies, and the diverse methodological choices undertaken when carrying out an LCA, it is difficult to compare the results found in this study with those presented by other researchers. However, most researchers agree that the environmental impact generated in the PV panel recycling process is always lower than that obtained from the equivalent production of the materials from virgin raw materials. Despite the difficulty of conducting a comparative analysis, the results and conclusions reached by various researchers in the study of the environmental impact of different PV panel recycling technologies are detailed below.

Latunussa et al. (2016)^14^ applied LCA to a recycling process for silicon PV panels based on mechanical and thermal treatments followed by acid leaching and electrolysis. The environmental benefits derived from the production of secondary raw materials were not considered, which in our study were demonstrated to be crucial. Most of the impact occurred during the incineration of the encapsulation and backsheet layers, followed by the treatments to recover metallic silicon, silver, copper, and aluminum. The authors highlighted the difficulty in comparing these results with those from other research due to the lack of a single recycling process and the undetailed data often provided, as we also noted.

Aryan et al. (2018)^50^ compared the environmental impacts of incinerating or pyrolyzing fluorinated and fluorine-free backsheet material from PV modules. The fluorine-free material showed better environmental performance than that of the fluorinated material for both thermal treatments for most impact categories. In our study, the fluorinated species released during pyrolysis also contributed significantly to the environmental impact. The authors also recommended the use of alkaline reagents and subsequent treatment of the resulting effluents to deal with the large quantities of hydrogen fluoride and halogenated aromatic hydrocarbons generated during pyrolysis. Additionally, pyrolysis products, such as oil and char, contained high amounts of fluorine, which made them difficult to use.

Song (2024)^51^ proposed two recycling scenarios for recovering silicon, copper, and silver from PV panels. The first scenario used a combination of thermal and mechanical treatments, similar to our study. This process was based on the mechanical separation of part of the glass, whereas the remainder underwent a thermal process. Unlike our study, the solid from the thermal process was treated by alkaline leaching and nitric acid to separate silicon from copper and silver. The second scenario used a pretreatment based on hydraulic fragmentation processes. Material recovery after pretreatment in both scenarios was achieved through hydrometallurgical processing. Their LCA results showed better performance in the second scenario, with improved results in all evaluated categories except for the global warming potential.

Duan et al. (2024)^52^ conducted an LCA of four scenarios of PV recycling: mechanical recycling, which included frame and connector removal, crushing, grinding with a turbine system, and separating the mixed polymers from the silicon and silver mixture; thermal recycling, which included separating the frame and connectors, cutting the panel, heating it in a furnace to separate the polymers, cooling it, separating the glass, and performing a hydrometallurgical treatment to extract the silicon and aluminum fragments; chemical recycling, which included separating the frames and connectors, using toluene at 90 °C for separation, recovering the glass and backsheets, and performing a hydrometallurgical treatment on the remaining components to extract silicon, silver, and aluminum; and green solvent recycling with DMI and DES. They found out that recycling with green solvents had a lower environmental impact. The main cause of the environmental impact of mechanical recycling is electricity consumption, while both electricity consumption and exhaust emissions are the main factors in thermal recycling, as in our study. Key advantages of our recycling method include glass removal prior to the thermal treatment, which reduces energy consumption, and not using solvents that would subsequently need to be recovered or recycled.

Finally, Wang et al. (2026)^53^ studied the recycling of PV modules, including transportation, manual dismantling, material separation, and final treatment. Out of the treatment systems analyzed, pyrolysis generated the largest carbon emissions, followed by HCl leaching, NaOH leaching, gas–solid fluidized bed processing, and dense media cyclone separation. In contrast, the recovery of aluminum frames and junction boxes yielded net environmental benefits, as in our study. The authors proposed a recycling route that included manual removal of aluminum frames and junction boxes, pyrolysis for EVA removal, separation of silicon and glass using a dense media cyclone, treatment with NaOH to remove the aluminum back layer, selective silver extraction with HNO_3_, and removal of the silicon nitride antireflective coating with an HF-HNO_3_ acid mixture.

## 4 Conclusions

This study provides a comprehensive LCA of an industrial process for recycling EoL crystalline-silicon PV modules. A hotspot analysis revealed that the thermal section is responsible for over 61.7% of the total environmental impact, while 38.3% is attributed to the mechanical section. This is due to the large values obtained for photochemical ozone formation and climate change, mostly caused by the thermal section, which dominates the single score. In contrast, the values for the remaining impact categories are allocated mostly to the mechanical section, with 54.2–77.3% of the impact in each impact category. 49.3% of the total environmental impact is caused by the significant electricity usage, 21.9% of which was consumed by the impact turbine mill and 21.7% by the pyrolysis oven. Consequently, this study proposed ways to optimize the energy use of the electricity-powered equipment, particularly that of the pyrolysis oven, for example, by using alternative heating methods and PV electricity.

Subtracting the impact caused by the avoided products results in negative values for all impact categories, proving the benefits of recycling PV modules. Recycling and/or reusing these materials, particularly silicon from metallic powder, cables, and junction boxes, is highly beneficial environmentally, mostly for the use of mineral and metal resources and water use impact categories.

The superior environmental performance of integrated mechanical-thermal recycling compared with mechanical recycling alone has also been demonstrated. The impact value is lower in all categories. Overall, the single score for the integrated recycling system is 23.1% lower than that for the mechanical recycling system alone.

In conclusion, this study provides one of the first LCAs of an integrated mechanical-thermal recycling process for PV waste on an industrial scale. The results advance the understanding of the feasibility of large-scale recycling and can be used to inform evidence-based strategies for the sustainable and circular management of PV waste. Ultimately, this research contributes to bridging the gap between theoretical recycling models and practical, scalable solutions that uphold sustainability principles. This reinforces not only the role of recycling as a key component in the transition toward a low-carbon, resource-efficient society but also the role of PV energy in the current energy transition, which aims to replace all fossil-based energy with greener alternatives.
